# Effectiveness of acupuncture combined mecobalamin in the treatment of elderly diabetic peripheral neuropathy: A protocol of systematic review and meta-analysis

**DOI:** 10.1097/MD.0000000000020366

**Published:** 2020-06-05

**Authors:** Yu-hong Duan, Ai-xia Liu, Hong-xia Su, Ji-hong Lv, Xue-ying Gong, Li Wang

**Affiliations:** Second Ward of Endocrinology Department, Affiliated Hospital of Shaanxi University of Chinese Medicine, Xianyang, China.

**Keywords:** acupuncture, diabetic peripheral neuropathy, effectiveness, mecobalamin, safety

## Abstract

**Background:**

Although previous studies have reported the effectiveness of acupuncture combined mecobalamin (AM) in the treatment of elderly diabetic peripheral neuropathy (EDPN), no systematic study has assessed its effectiveness and safety. Thus, this study will evaluate the effectiveness and safety of AM for the treatment of patients with EDPN.

**Methods:**

Bibliographic electronic databases will be searched as follows: Cochrane Library, PUBMED, EMBASE, CINAHL, PsycINFO, WANGFANG, and China National Knowledge Infrastructure. All of them will be searched from each database initial to March 1, 2020 without language restrictions. All study selection, information extracted, and study quality evaluation will be performed by 2 independent authors. Any disagreements between 2 authors will be resolved by a third author via discussion. RevMan 5.3 software will be used for data pooling and meta-analysis performance if it is possible.

**Results:**

This study will provide synthesis of current evidence of AM for patients with EDPN through primary outcome of glycemic profile, and secondary of neuropathic pain intensity, plantar tactile sensitivity, sensory nerve conduction velocity and motor nerve conduction velocity, health-related quality of life, and adverse events.

**Conclusion:**

This study will provide helpful reference for the efficacy and safety of AM for the treatment of patients with EDPN to the clinicians and further studies.

**Study registration number:** INPLASY202040094.

## 1 Introduction

Elderly diabetic peripheral neuropathy (EDPN) is one of the most severe chronic microvascular complications in patients with diabetes mellitus (DM),^[1–5]^ which affects about 50% of DM patients.^[6–7]^ It manifests as high morbidity of neuropathic pain, foot ulceration, and amputation.^[8–11]^ The pathogenesis of EDPN is very complex and is still unclear up to the present.^[12–15]^ Currently, there is still not specific and highly effective pharmacologic curative approach for EDPN.^[16–20]^ Previous studies have reported that acupuncture combined mecobalamin (AM) can effectively treat patients EDPM.^[21–24]^ However, there is insufficient evidence to support the effectiveness and safety of AM for EDPN. The objective of this study is to carry out a systematic review of the literatures concerning the effectiveness and safety of AM for the treatment of EDPN.

## 2 Methods

### 2.1 Study registration

This study has been registered on INPLASY202040094. It has been reported according to the guideline of Preferred Reporting Items for Systematic Reviews and Meta-Analysis Protocol statement.^[25]^

### 2.2 Eligibility criteria

#### 2.2.1 Type of studies

Any randomized controlled trials (RCTs) exploring the effectiveness and safety of AM for the treatment of patients with EDPN will be included. We will not consider other studies, such as non-clinical trials, non-controlled trials, and non-RCTs.

#### 2.2.2 Type of participants

Studies on adult patients, 65 years old or above, who were diagnosed as EDPN will be included in this study. No limitations of location, educational background, and gender will be imposed.

#### 2.2.3 Type of interventions

Any forms of AM therapy used to treat patients with EDPN will be included in the experimental group.

Any other treatments, but not AM, used to manage participants with EDPN will be entered in the control group.

#### 2.2.4 Type of outcomes

The primary outcome includes glycemic profile, as measured by fasting blood glucose or glycated hemoglobin.

The secondary outcomes consist of neuropathic pain intensity, as assessed by visual analogue scale or other relevant tools; plantar tactile sensitivity, as evaluated by Semmes-Weinstein monofilament; sensory nerve conduction velocity and motor nerve conduction velocity, as checked by electromyography; quality of life, as evaluated by Health-Related Quality of Life scale or associated scores; and adverse events.

### 2.3 Search strategy

We will perform searches via the bibliographic electronic databases of Cochrane Library, PUBMED, EMBASE, CINAHL, PsycINFO, WANGFANG, and China National Knowledge Infrastructure. We will search all those databases from inception to March 1, 2020 with no restrictions of language and publication status. The search terms are diabetic neuropathy, peripheral neuropathy, neuropathy, diabetic, diabetic polyneuropathy, diabetic neuropathies, diabetes mellitus, elderly, acupuncture, acupuncture therapy, acupuncture ear, auriculotherpay, electroacupuncture, acupoint, and mecobalamin. The sample of search strategy for PUBMED is presented in Table 1. Similar search strategies for other electronic databases will be adapted and applied.

**Table 1:**
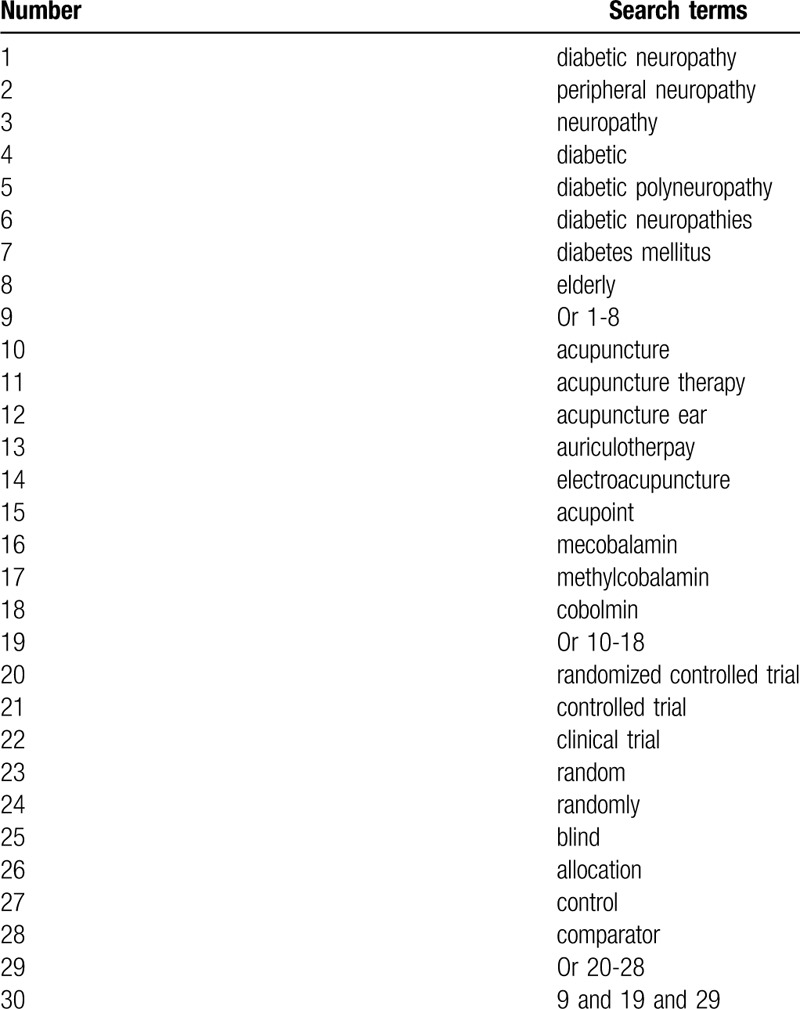
Search strategy for PUBMED.

In addition, we will identify conference abstracts, dissertations, and reference lists of relevant reviews.

### 2.4 Data selection and extraction

#### 2.4.1 Study selection

All searched records will be imported to the Endnote 7.0, and we will de-duplicate all irrelevant references. All titles and abstracts will be scanned independently by 2 of the authors in consideration of the eligibility criteria. A third author will be asked to help reaching a general decision in case of any divergences between 2 authors. After that, the full-text of all potential studies will be checked for further screening. We will record all removed studies with specific reasons. The process of study selection will be shown in a flowchart.

#### 2.4.2 Data extraction

Before data collection, a data extraction sheet will be built by our study team. Two authors will separately collect relevant information from each eligible study. Any discrepancies will be handled by discussion and consultation with a third experienced author. The following items will be extracted: study title, first author, year of publication, number of patients in different groups, age, gender, course of EDPN, study design, study setting, study duration, types of interventions, controls, dosage, all endpoints, safety, funding information, and any other relevant information.

### 2.5 Study quality assessment

The study quality assessment will be done using Cochrane Risk of Bias Tool, which contains 7 domains. The outcome of each domain will be classified as high, unclear, and low risk of bias. The whole process will be performed by 2 authors, and inconsistence will be solved by consultation with a third author.

### 2.6 Data synthesis and analysis

ReMan 5.3 software is used for data synthesis and meta-analysis if it is possible. Mean difference or standardized mean difference and 95% confidence intervals (CIs) will be used to calculate quantitative data, and dichotomous data will be exerted as risk ratio and 95% CIs. Statistical heterogeneity across studies was done with *I*^*2*^ statistic. *I*^*2*^ ≤ 50 indicates homogeneity among studies, and a fixed-effects model will be employed for pooled analysis. *I*^*2*^ > 50% suggests obvious heterogeneity, and a random-effects model will be employed for synthesized analysis. When there is homogeneity of the merged outcome results across sufficient studies, meta-analysis will be conducted. Otherwise, we will carry out subgroup analysis to explore causes of obvious heterogeneity. We will report a narrative synthesis using detailed written commentary on the different study characteristics (such as location, and duration), patient characteristics (such as gender, and course of EDPN), different interventions and controls (such as dosage, and frequency), and outcome measurements.

### 2.7 Subgroup analysis

If necessary, subgroup analysis will be conducted based on the different study qualities, interventions, controls and outcome measurements.

### 2.8 Sensitivity analysis

Sensitivity analysis will be undertake to check the stability of merged outcome results by excluding studies with high risk of bias.

### 2.9 Reporting bias

Funnel plot^[26]^ and Egger regression test^[27]^ will be checked to find potential reporting bias if sufficient studies are included.

### 2.10 Ethics and dissemination

No ethic approval is inquired, because this study will based on the data of published literature. This study is expected to be published at a peer-reviewed journal.

## 3 Discussion

EDPN is very common complication in diabetes patients, especially among the elderly population. Currently, medication management is widely used for this condition; however, there are still some shortcomings, such as limited effectiveness and severe side effects. Thus, more effective managements with fewer alternative therapies are urgently needed, such as AM. Previous studies have reported that AM can benefit for patients with EDPN. However, there is no systematic review to explore this issue. Thus, this study is the first one to investigate the effectiveness and safety of AM for the treatment of patient with EDPN systematically. The results of this study will provide helpful evidence for both clinical practice and future studies.

## Author contributions

**Conceptualization:** Yu-hong Duan, Hong-xia Su, Xue-ying Gong, Li Wang.

**Data curation:** Yu-hong Duan, Ai-xia Liu, Hong-xia Su, Xue-ying Gong, Li Wang.

**Formal analysis:** Yu-hong Duan, Ji-hong Lv, Li Wang.

**Investigation:** Ai-xia Liu, Hong-xia Su.

**Methodology:** Yu-hong Duan, Ji-hong Lv, Xue-ying Gong.

**Project administration:** Hong-xia Su.

**Resources:** Yu-hong Duan, Ai-xia Liu, Ji-hong Lv, Xue-ying Gong, Li Wang.

**Software:** Yu-hong Duan, Ai-xia Liu, Ji-hong Lv, Xue-ying Gong, Li Wang.

**Supervision:** Hong-xia Su.

**Validation:** Yu-hong Duan, Ai-xia Liu, Hong-xia Su, Ji-hong Lv, Xue-ying Gong, Li Wang.

**Visualization:** Yu-hong Duan, Hong-xia Su, Li Wang.

**Writing – original draft:** Yu-hong Duan, Ai-xia Liu, Hong-xia Su, Xue-ying Gong.

**Writing – review & editing:** Yu-hong Duan, Ai-xia Liu, Hong-xia Su, Ji-hong Lv, Xue-ying Gong, Li Wang.
